# Comparing Adductor Canal Blocks With Bupivacaine and Magnesium to Bupivacaine and Buprenorphine After Same-Day Discharge Total Knee Arthroplasty: A Prospective Randomized Controlled Trial

**DOI:** 10.7759/cureus.86492

**Published:** 2025-06-21

**Authors:** Nazia Siddiqui, Kinjal Patel, Ronak Desai, Joffer Hakim, Farhad Ghoddoussi, Paul Mah, Matthew Cao, Vidhi Patel, Hannah Roop, Sandeep Krishnan

**Affiliations:** 1 Anesthesiology, Wayne State University School of Medicine, Detroit, USA; 2 Anesthesiology, Cooper University Hospital, Camden, USA; 3 Anesthesiology, Westchester Medical Center, Valhalla, USA; 4 Anesthesiology, Johns Hopkins University School of Medicine, Baltimore, USA

**Keywords:** adductor canal block, adjuvant, buprenorphine, magnesium sulfate, same-day discharge total knee arthroplasty, total knee arthroplasty

## Abstract

Objectives

Same-day discharge (SDD) total knee arthroplasty (TKA) can provide significant cost savings when compared to inpatient TKA. Advances in surgical techniques, early mobilization, and perioperative pain control have been the primary facilitators of this move toward SDD. Using adjuvant medications, such as magnesium and buprenorphine, along with local anesthetics in regional anesthetic techniques has been shown to be effective in prolonging analgesic effects. The objective of this study was to compare the effects of using magnesium and bupivacaine to buprenorphine and bupivacaine in adductor canal blocks (ACB) on postoperative pain, opioid consumption, nausea, and overall satisfaction.

Methods

A total of 105 adults undergoing elective unilateral SDD TKA were included. An a priori power analysis was conducted using G*Power 3.1.6 (Heinrich Heine University Düsseldorf, Düsseldorf, Germany) to calculate sample size. Patients were randomly assigned to receive ACB with magnesium and bupivacaine (n = 62) or buprenorphine and bupivacaine (n = 43). Primary outcomes were opioid consumption and pain scores for the first 48 hours after surgery. Secondary outcomes included the incidence of nausea in the first 48 hours after surgery and overall satisfaction with the surgical experience measured using a Likert scale from 0 to 10.

Results

There was no significant difference in pain scores in the magnesium group compared to the buprenorphine group over the first 24 hours (4.1 ± 1.8 vs. 3.7 ± 1.9, P = 0.375) and the second 24 hours (4.4 ± 2.2 vs. 4.2 ± 1.9, P = 0.637) after surgery. The difference in opioid consumption was also nonsignificant when comparing the magnesium group to the buprenorphine group over the first 24 hours (61.4 ± 50.6 vs. 47.4 ± 39.2, P = 0.108), the second 24 hours (33.0 ± 40.1 vs. 21.7 ± 20.8, P = 0.590), and the first 48 hours (28.0 ± 19.9 vs. 25.7 ± 21.2, P = 0.148) total after surgery. Secondary outcomes showed no difference in the incidence of nausea over the first 48 hours and overall satisfaction.

Conclusion

Magnesium and buprenorphine are comparable as adjuvants in their effects on postoperative pain and opioid consumption at 24 and 48 hours after SDD TKA, with similar incidences of nausea and vomiting.

## Introduction

Recent advances in surgical techniques, as well as perioperative rapid recovery protocols, have been effective in reducing average length of stay (LOS) after total knee arthroplasty (TKA) [1]. In 2022, the average postoperative LOS after TKA was 1.3 days, a reduction of more than a day when compared to TKA LOS data from 2012 to 2021 [2]. Same-day discharge (SDD) TKA has been shown to yield a 30% median cost savings when compared to inpatient TKA. This cost saving is primarily related to inpatient surgical postoperative care, including pharmacy costs [3]. As the demand for TKA increases in an aging population, with projections of 1.26 million primary TKAs per year by 2030 [4], there is a push for optimization of hospital costs while maintaining or increasing quality and minimizing risk.

Improvements in surgical technique, early mobilization, and advances in perioperative pain control have been the primary facilitators of SDD TKA. The most common patient concern in studies on perceptions of the safety of SDD TKA was pain [5]. Historically, opioids have been the mainstay of perioperative pain control after TKA. However, to reduce postoperative opioid consumption after TKA, multimodal analgesic protocols, including regional anesthetic techniques, have become prevalent. Regional anesthesia has resulted in increased patient satisfaction, decreased LOS, and decreased opioid consumption along with associated decreases in opioid-related side effects [6]. Adductor canal block (ACB) is a widely used regional technique for the management of postoperative pain after TKA that allows for early ambulation and satisfactory pain control in the perioperative period [7,8].

Several adjuvant medications to local anesthetics have been investigated in an effort to prolong the analgesic effects of ACB. These include dexamethasone, dexmedetomidine, clonidine, epinephrine, magnesium, midazolam, tramadol, buprenorphine, fentanyl, and morphine [9]. Buprenorphine is a synthetic opioid that is a partial agonist at the mu receptor, an agonist at the delta receptor, and a weak antagonist at the kappa receptor. It is thought to act primarily on peripheral nervous system receptors when injected peripherally at the site of a nerve [10,11]. Magnesium sulfate is an N-methyl-D-aspartate (NMDA) receptor antagonist. It is thought to exert its analgesic effects in multiple ways. Magnesium blocks NMDA receptors and thereby acts as a physiological calcium antagonist. It also inhibits the inflammatory response by reducing inflammatory cytokines [12,13]. Both of these drugs have been previously studied as adjuvants to local anesthetics for regional anesthesia; however, there have been no studies to date comparing the addition of magnesium or buprenorphine to local anesthetics for ACBs after SDD TKA.

The primary objectives of this study were to compare the effects of magnesium and bupivacaine to buprenorphine and bupivacaine in ACBs on pain scores and opioid consumption in the first 48 hours after SDD TKA. The secondary outcomes were to examine the incidence of nausea and vomiting and to observe overall satisfaction with the SDD TKA process.

## Materials and methods

Study design and ethics

This prospective, randomized, double-blinded, controlled study was conducted by the Department of Anesthesiology at Trinity Oakland Hospital in Pontiac, Michigan, USA. Institutional Review Board approval was obtained from the Trinity Oakland Institutional Review Board (Approval No.: 2021-058). The study was registered at clinicaltrials.gov (NCT05091138). The investigators enrolled 105 consecutive patients between April 2022 and July 2023. All study patients signed informed written consent in the preoperative holding area prior to surgery.

Participants

Eligibility Criteria

Patients older than 18 years undergoing elective SDD unilateral TKA were eligible for enrollment. Patients with known clotting or bleeding disorders, taking anticoagulants or anti-platelet agents (without stopping them appropriately), with previous lumbar or lower extremity surgery that prevented spinal or regional anesthesia, with neuropathy or pre-existing neurological deficits, or who were chronic opioid users were excluded from the study.

Study procedures

Patients received oral celecoxib (200 mg) and oral acetaminophen (1 g) as part of our institutional multimodal protocol one hour prior to surgery (unless contraindicated due to allergy/medical condition). Patients deemed clinically appropriate for benzodiazepines were premedicated with 2 mg midazolam, and 3 mL (60 mg) of preservative-free isobaric 2% mepivacaine was administered via intrathecal injection. A target-controlled infusion pump was used to deliver a propofol infusion (30-60 mcg/kg/min and titrated to maintain adequate sedation) for maintenance of anesthesia. Sedation was monitored using standard clinical parameters at the discretion of the anesthesiology team; bispectral index and entropy monitoring were not used. The orthopedic surgeon administered an intra-articular injection of 20 mL of 0.25% ropivacaine and 30 mg of ketorolac while still in the operating room immediately following surgery. Opioid medications and other multimodal analgesics, including ketamine, dexmedetomidine, and acetaminophen, were not administered (oral or parenteral) during the intraoperative period. However, 4 mg of dexamethasone was given parenterally to all patients during surgery for postoperative nausea and vomiting prophylaxis.

The patients were assigned to one of two groups based on a computer-generated randomization chart. The surgeon, intraoperative anesthesiology staff, and patients were blinded to group assignment. Patients in group A (Bup) received a postoperative ACB containing 30 mL of 0.25% bupivacaine and 300 mcg (1 mL) of buprenorphine. Patients in group B (Mag) received a postoperative ACB containing 30 mL of 0.25% bupivacaine, 150 mg (0.3 mL) of magnesium sulfate, and 1.7 mL of sterile saline.

**Figure 1:**
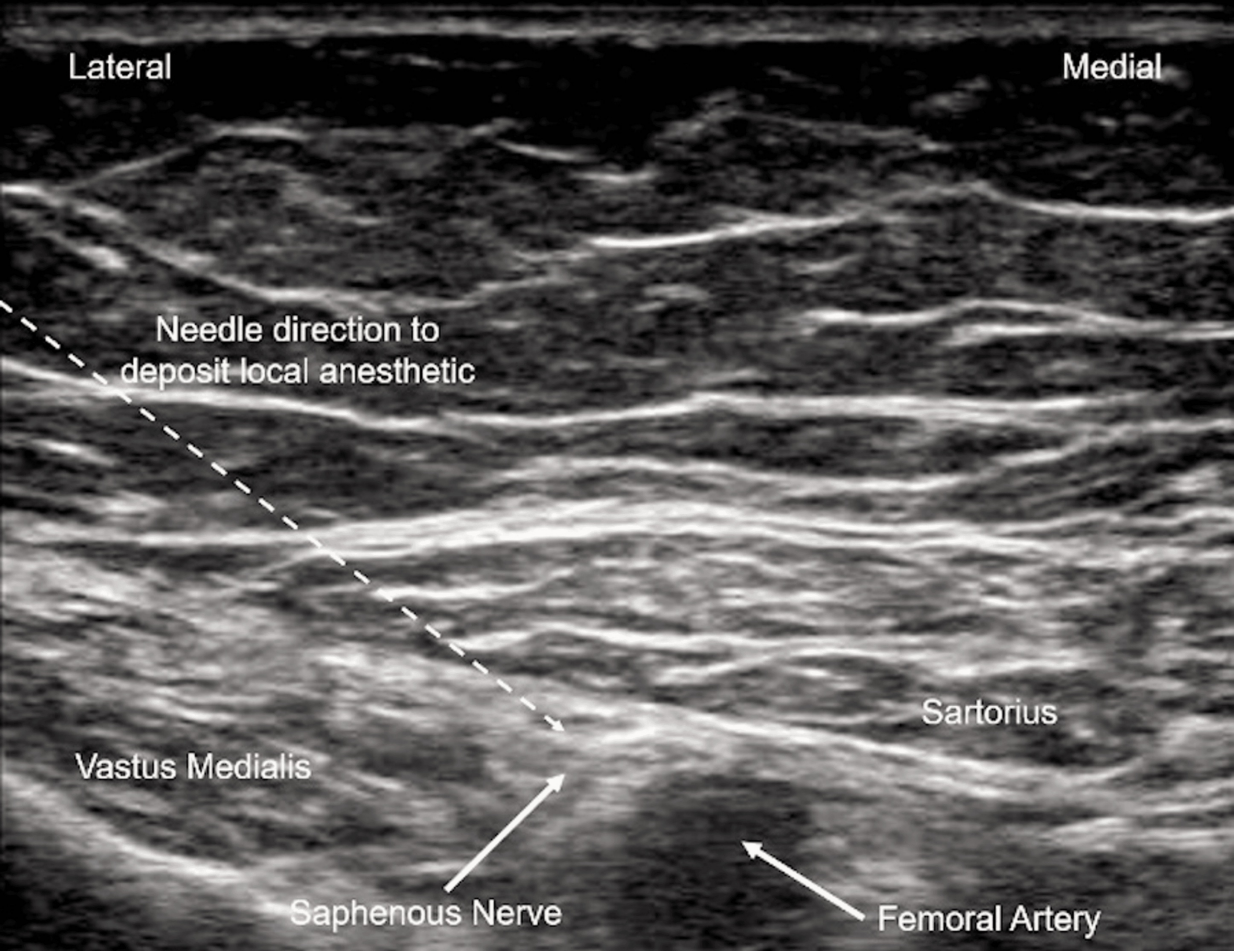
Ultrasound image showing needle direction and anatomical location for depositing local anesthetic for adductor canal block (ACB).

**Figure 2:**
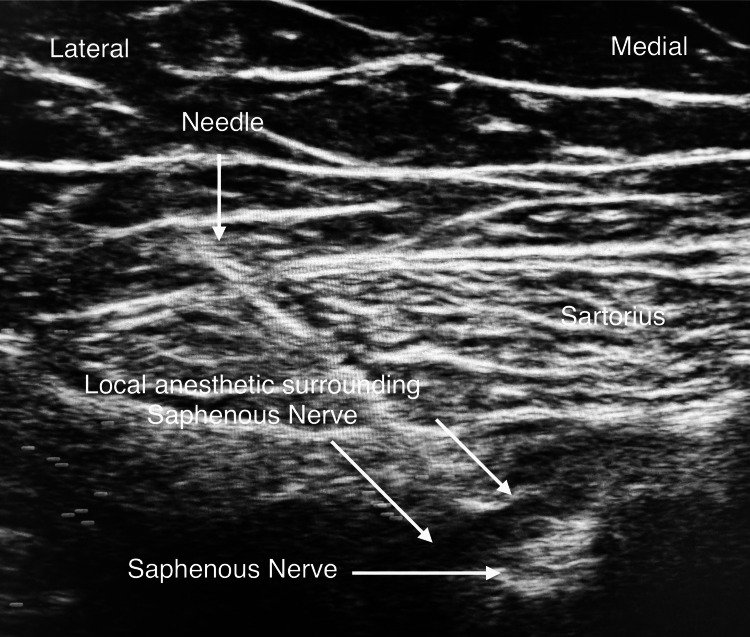
Ultrasound image showing needle deposition of local anesthetic around the saphenous nerve for adductor canal block (ACB).

All ACBs were performed using ultrasound in the anesthesia recovery area by senior anesthesiology residents and supervised by one of two senior anesthesiologists. The patient’s operative leg was abducted and externally rotated, and a linear ultrasound probe was placed in the mid-thigh region. After the adductor canal was identified, a needle was inserted in close proximity to the saphenous nerve, and local anesthetic solution was injected under direct visualization (Figures 1, 2).

The primary anesthesiologist met with all patients prior to discharge home. A data sheet was provided (Appendix A) to each patient to record pain scores (using a visual analog scale (VAS)), opioid use, incidence of nausea, and overall satisfaction at 24 hours and 48 hours post surgery. A survey was sent via text message or email to each patient (Appendix B). The surveys were completed online, and the data were transmitted directly to a spreadsheet.

Choice of postoperative opioid prescription was left to the orthopedic team and patient preference. Patients were allowed to use acetaminophen and ibuprofen on an as-needed basis for non-severe pain, when clinically appropriate and not contraindicated. Opioid consumption for all patients was converted to oral morphine equivalents (OME) based on an opioid conversion calculator (http://www.globalrph.com/narcotic.cgi).

Statistical analysis

The primary outcomes of the investigation were total opioid consumption (OME, mg) for the first 24 hours (day one), the second 24 hours (day two) post surgery, and both days combined (total). Pain scores were measured at 24 hours (day one) and 48 hours (day two) post surgery. Secondary outcomes included overall patient satisfaction at 24 hours (day one) and 48 hours (day two) after surgery, and the incidence (percentage of the patients) of nausea or vomiting during the first 24 hours (day one) and the second 24 hours (day two) post surgery and combined (in either of the two days). All outcomes were analyzed as continuous variables except for the incidence of nausea, which was treated as a categorical variable.

Demographic data were analyzed using t tests, χ2 tests, and Fisher's exact tests, as appropriate. The statistical differences between the Bup and Mag groups, for the continuous data, were determined using a one-way analysis of variance (ANOVA). For the categorical data, the statistical difference was determined using Pearson's χ2, followed by Yates's continuity correction and Fisher's exact probability test. A p-value of less than 0.05 was considered statistically significant. A p-value of 0.05 < p < 0.1 (significant one-tail) can indicate a trend that could become significant with stronger power in the study. Data are reported as numbers (% of patients), mean ± standard error of the mean, or mean ± standard deviation. All the data were analyzed using IBM SPSS version 24 (IBM Corp., Armonk, NY).

## Results

**Figure 3:**
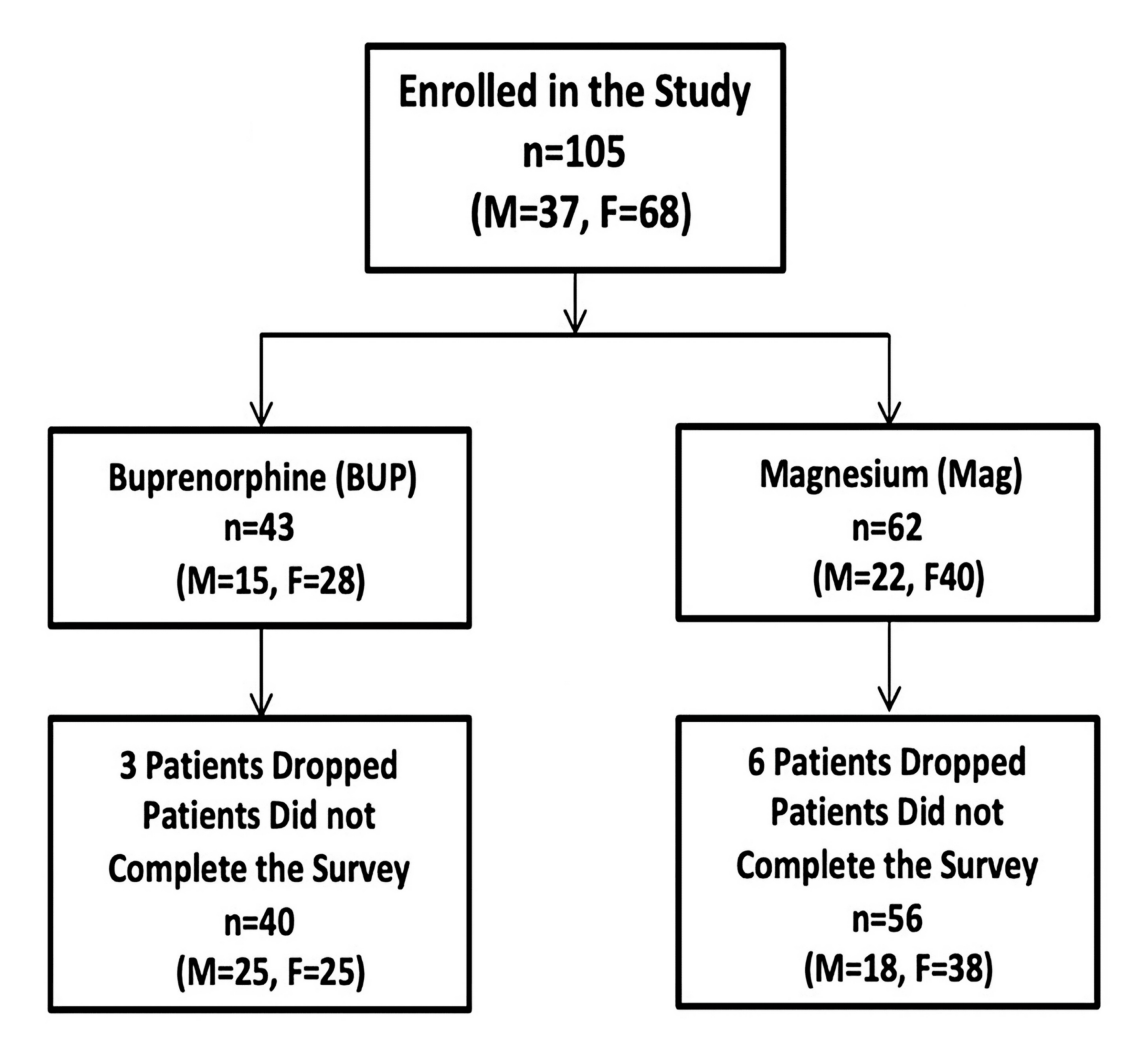
Consolidated Standards of Reporting Trials (CONSORT) diagram.

Figure 3 displays the Consolidated Standards of Reporting Trials (CONSORT) diagram for this study. A total of 105 patients were enrolled, 43 in the Bup group and 62 in the Mag group. Nine patients did not return the surveys and, therefore, were excluded from the study, resulting in a total of 96 patients (Bup = 40 and Mag = 56).

**Figure 4:**
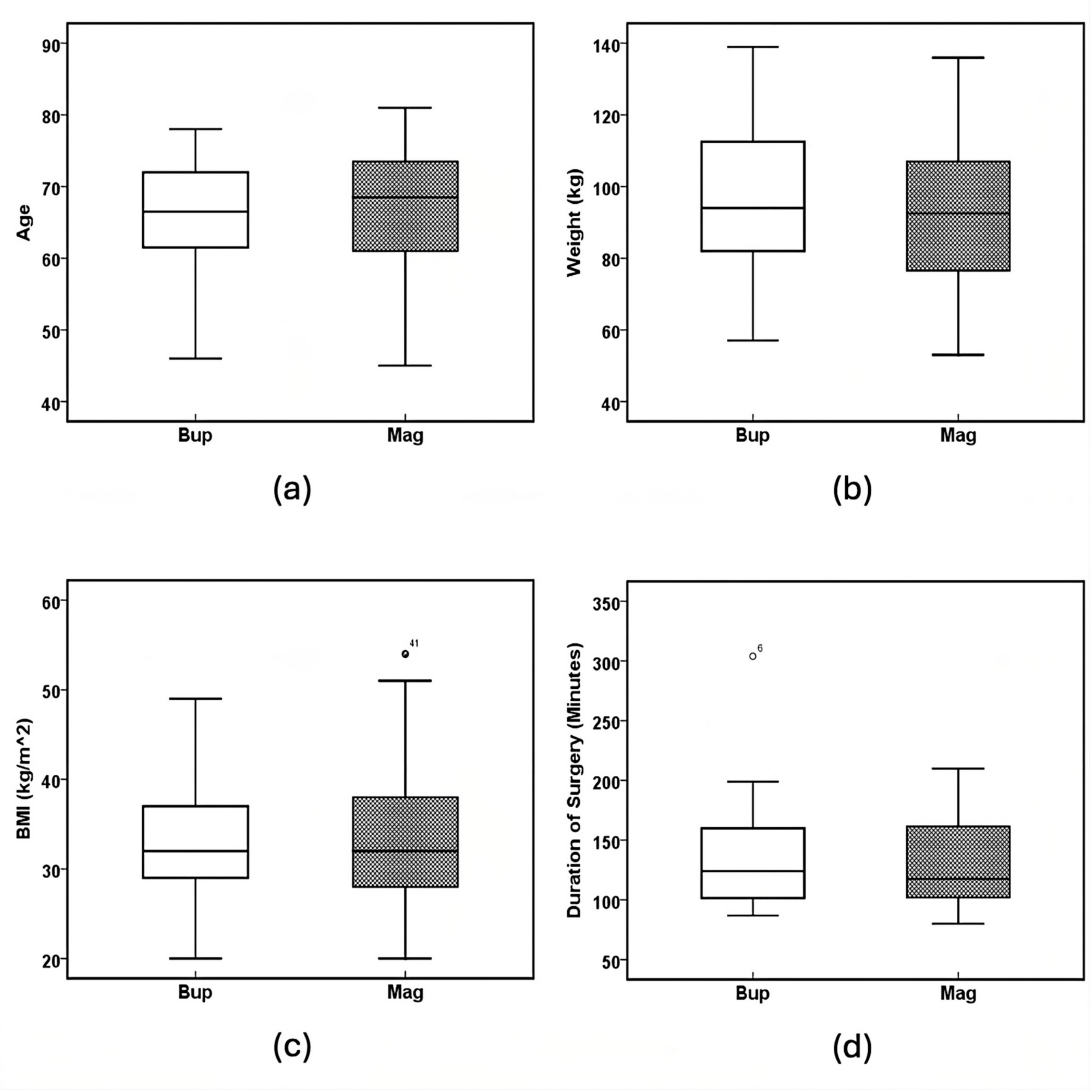
Patient characteristics. Boxplots of (a) age (years) (p = 0.319), (b) weight (kg) (p = 0.444), (c) BMI (kg/m^2^) (p = 0.937), and (d) duration of surgery (min) (p = 0.677). The solid line in the middle of the box represents the median. The box represents the middle 50%, and the whiskers represent the top and bottom 25%. Mild outliers are represented by circles and extreme outliers by stars. Bup: buprenorphine; Mag: magnesium.

There was no significant difference between the Bup and Mag groups with respect to gender (Pearson's X^2^ = 0.3, p = 0.584, Fisher's exact probability test, two-tailed p = 0.664, Table 1), race (Fisher's exact probability test, two tailed p = 0.655, Table 1), age (F(1, 94) = 1.004, p = 0.319, Table 1 and Figure 4B), weight (F(1, 94) = 0.591, p = 0.444, Table 1 and Figure 4A), BMI (F(1, 94) = 0.006, p = 0.937, Table 1, and Figure 4C), American Society of Anesthesiologists (ASA) grade (Fisher's exact probability test, two-tailed p = 0.678, Table 1), and the duration of surgery (min) (F(1, 94) = 0.174, p = 0.677, Table 1 and Figure 4D).

**Table 1:** Patient demographics and perioperative data. Values are mean ± SD or number (% of patients). * P-value < 0.05 is considered statistically significant. # P-value 0.05 < p < 0.1 is significant in a one-tail test. ASA: American Society of Anesthesiologists.

	Buprenorphine	Magnesium	P-values
	n = 40	n = 56	
Gender			
Male	15 (37.5)	18 (32)	0.664
Female	25 (62.5)	38 (68)	
Race			
Asian	1 (2.5)	0 (0)	0.655
Black	2 (5)	2 (4)	
Hispanic	1 (2.5)	2 (4)	
White	36 (90)	52 (92)	
Age (years)	65.3 ± 8.0	66.9 ± 7.9	0.319
Weight (kg)	96.4 ± 19.9	93.2 ± 20.9	0.444
BMI (kg/m^2^)	32.9 ± 6.8	33.0 ± 7.5	0.937
ASA I	0 (0)	0 (0)	0.678
ASA II	19 (47.5)	24 (43)	
ASA III	21 (52.5)	30 (54)	
ASA IV	0 (0)	2 (3)	
Duration of surgery (min)	133.8 ± 43.1	130.4 ± 36.2	0.677

**Figure 5:**
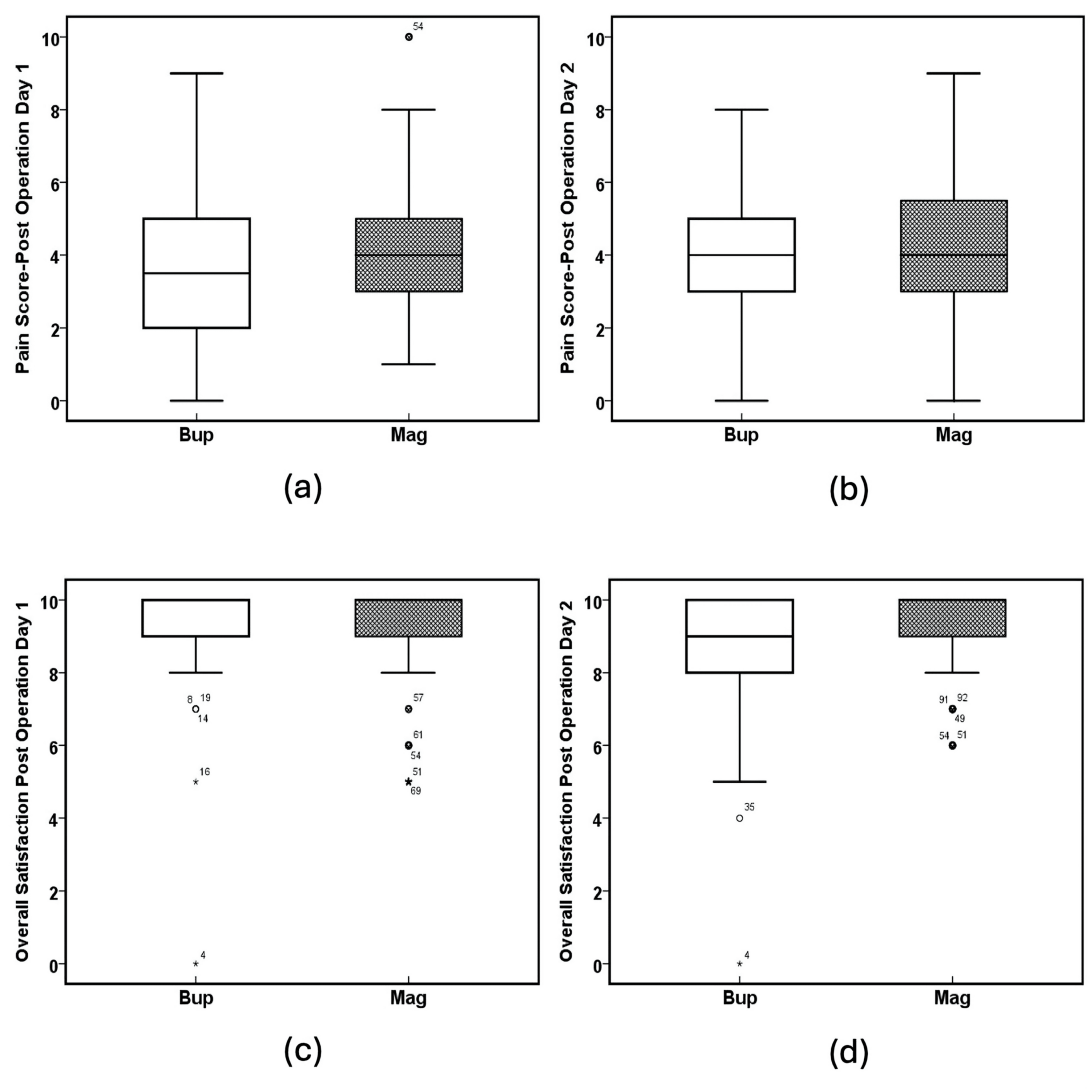
Pain scores and satisfaction. (a) Pain score on postoperative day one (p = 0.375), (b) pain score on postoperative day two (p = 0.637), (c) overall satisfaction on postoperative day one (p = 0.601) (scores 0 to 10, 10 = most satisfied), and (d) overall satisfaction on postoperative day two (p = 0.063) (scores 0 to 10, 10 = most satisfied). The solid line in the middle of the box represents the median. The box represents the middle 50%, and the whiskers represent the top and bottom 25%. Mild outliers are represented by circles and extreme outliers by stars. Bup: buprenorphine; Mag: magnesium.

ANOVA analysis showed there was no significant difference between the Bup and Mag groups with respect to pain score on postoperative day one (F(1, 94) = 0.795, p = 0.375, Table 2 and Figure 5A), pain score on postoperative day two (F(1, 94) = 0.224, p = 0.637, Table 2 and Figure 5B), and overall satisfaction on postoperative day one (F(1, 94) = 1.484, p = 0.601, Table 2 and Figure 5C). However, there was a potentially significant increase in the overall satisfaction on postoperative day two (F(1, 94) = 5.305, p = 0.063, Table 2 and Figure 5D) in the Mag group when compared to the Bup group.

**Table 2:** Patient outcome data. Values are mean ± SD or number (% of patients). * P-value < 0.05 is considered statistically significant. # P-value 0.05 < p < 0.1 is significant in a one-tail test.

	Buprenorphine	Magnesium	P-values
	n = 40	n = 56	
Pain score post-op., day 1	3.7 ± 1.9	4.1 ± 1.8	0.375
Pain score post-op., day 2	4.2 ± 1.9	4.4 ± 2.2	0.637
Overall satisfaction, day 1	9.2 ± 1.2	9.4 ± 1.3	0.601
Overall satisfaction, day 2	8.8 ± 1.6	9.3 ± 1.2	0.063

**Figure 6:**
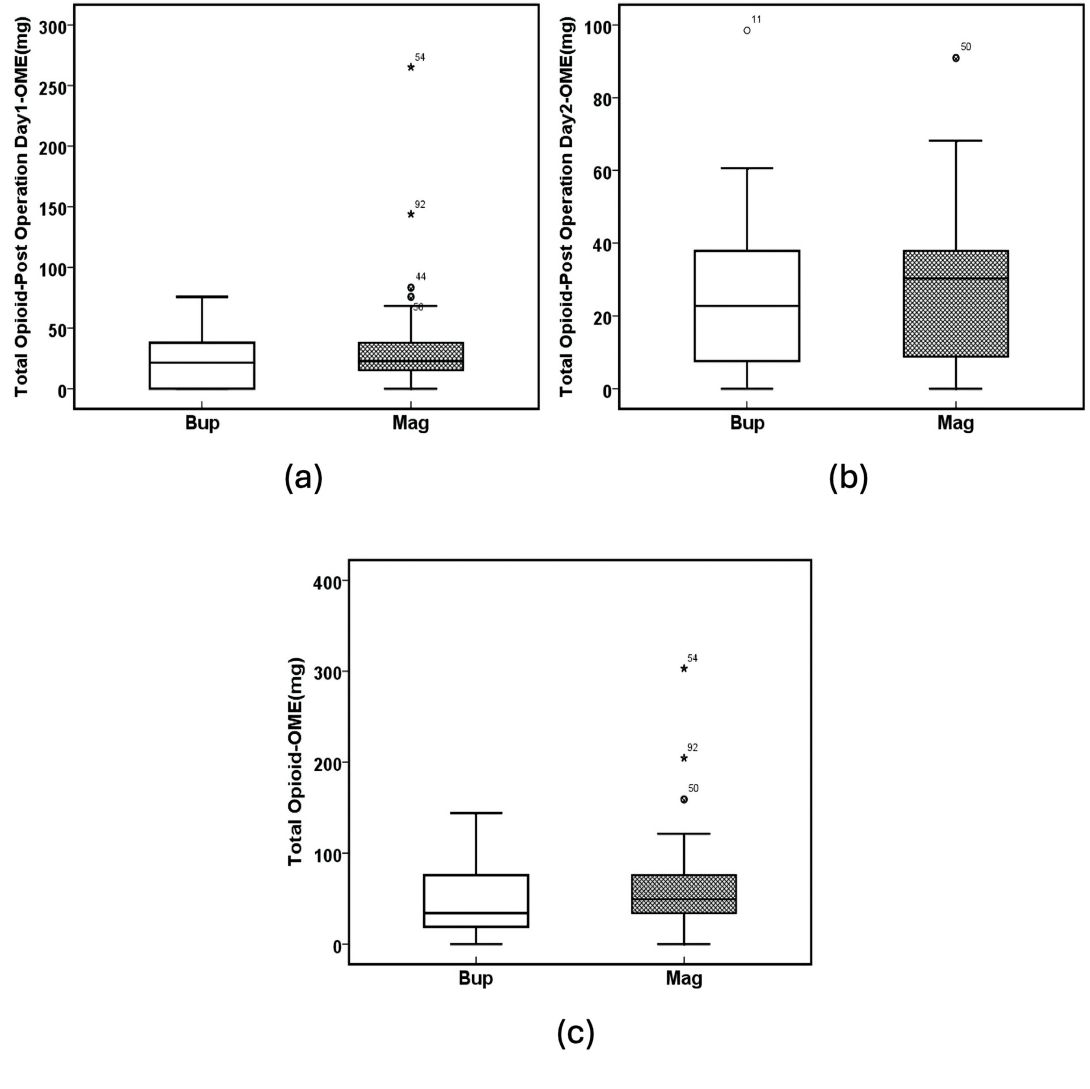
Opioid consumption. (a) Total opioid on postoperative day one - OME (mg) (p = 0.108), (b) total opioid on postoperative day two - OME (mg) (p = 0.590), and (c) total opioid - OME (mg) (p = 0.148). The solid line in the middle of the box represents the median. The box represents the middle 50%, and the whiskers represent the top and bottom 25%. Mild outliers are represented by circles and extreme outliers by stars. Bup: buprenorphine; Mag: magnesium; OME: oral morphine equivalents.

ANOVA analysis showed there was no significant difference between the Bup and Mag groups with respect to total opioid on postoperative day one (OME, mg) (F(1, 94) = 2.631, p = 0.108, Table 3 and Figure 6A), total opioid on postoperative day two (OME, mg) (F(1, 94) = 0.292, p = 0.590, Table 3 and Figure 6B), and total opioid (OME, mg) (F(1, 94) = 2.131, p = 0.148, Table 3 and Figure 6C).

**Table 3:** Medications used. Values are mean ± S.E. or number (% of patients). * P-value < 0.05 is considered statistically significant. # P-value 0.05 < p < 0.1 is significant in a one-tail test.

	Buprenorphine	Magnesium	P-values
	n = 40	n = 56	
Total opioid, oral morphine equivalents (mg)	47.4 ± 39.2	61.4 ± 50.6	0.108
Day 1 opioid, oral morphine equivalents (mg)	21.7 ± 20.8	33.0 ± 40.1	0.59
Day 2 opioid, oral morphine equivalents (mg)	25.7 ± 21.2	28.0 ± 19.9	0.148
Total # of patients with nausea and/or vomiting	Yes = 8 (20), No = 30 (75)	Yes = 12 (16), No = 41 (84)	1
Day 1 # of patients with nausea and/or vomiting	Yes = 5 (13), No = 34 (85)	Yes = 5 (9), No = 51 (91)	0.736
Day 2 # of patients with nausea and/or vomiting	Yes = 8 (20), No = 31 (78)	Yes = 12 (21), No = 41 (73)	1

**Figure 7:**
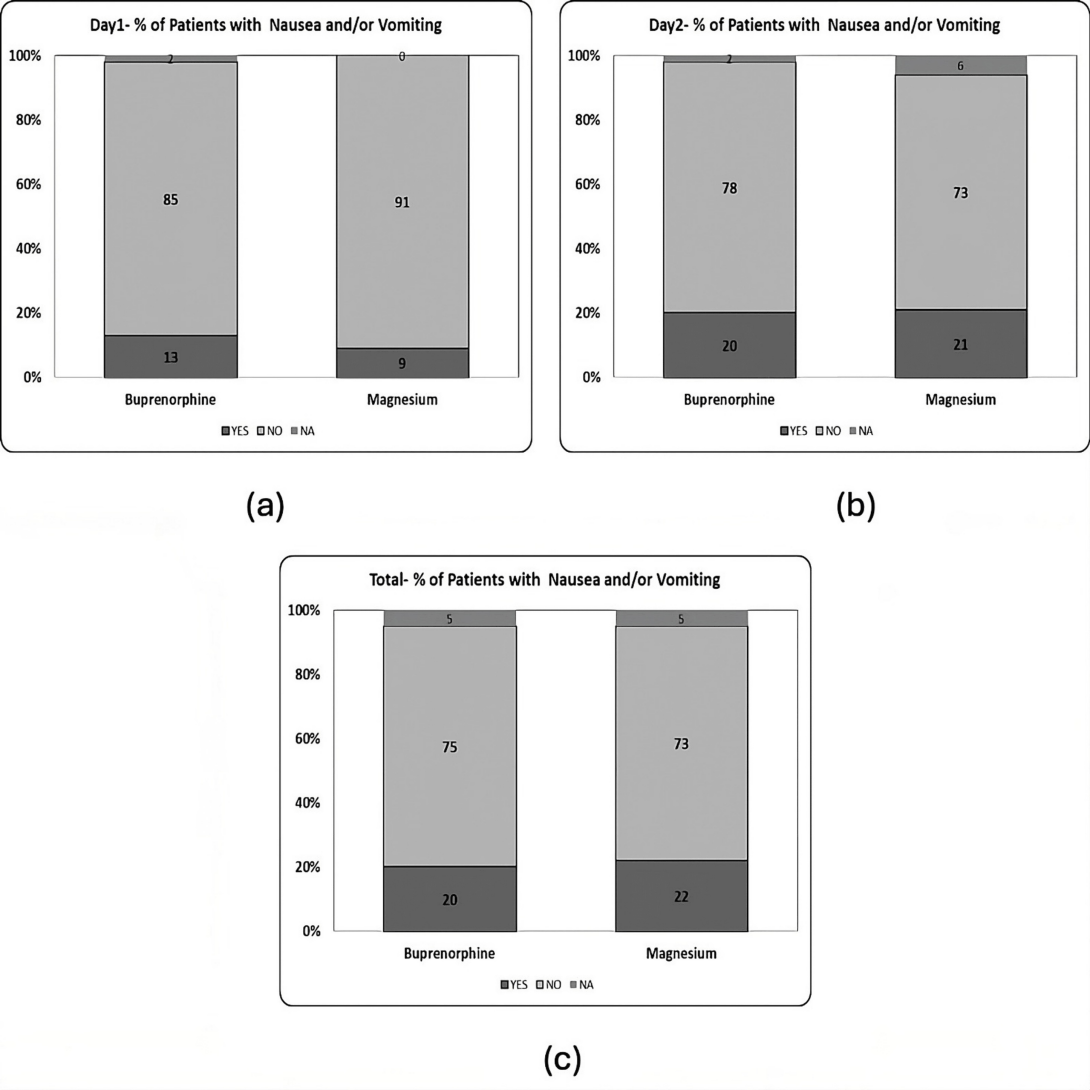
Nausea and vomiting. (a) Day one - % of patients with nausea and/or vomiting (p = 0.736), (b) day two - % of patients with nausea and/or vomiting (p = 1.000), and (c) total - % of patients with nausea and/or vomiting in either of the days (p = 1.000).

There was no significant difference in nausea or vomiting on postoperative day one between the Bup and Mag groups (Fisher's exact probability test, two-tailed p = 0.736, Table 3 and Figure 7A). This was also true on postoperative day two (Pearson's X^2 ^= 0.06, p = 0.806, Fisher's exact probability test, two-tailed p = 1, Table 3 and Figure 7B) and for the total number of patients with nausea and/or vomiting (Pearson's X^2 ^= 0.03, p = 0.862, Fisher's exact probability test, two-tailed p = 1, Table 3 and Figure 7C).

## Discussion

To date, this is the first trial to compare ACBs performed with buprenorphine as an adjuvant versus magnesium in SDD TKA. This prospective, controlled, randomized, double-blinded study demonstrates that both buprenorphine and magnesium have comparable analgesic effects when used as adjuvants to bupivacaine in ACBs for SDD TKA.

Regional anesthesia is a frequently used technique to provide analgesia after TKA. ACB has largely supplanted femoral nerve block (FNB) as the regional technique of choice for the management of postoperative pain after TKA. ACBs have demonstrated comparable analgesic effects to FNBs with relative sparing of quadriceps strength and mobilization [14]. Early ambulation after TKA is associated with reduced LOS, improved knee range of motion, and decreased postoperative pain [15]. Local anesthetics are used to facilitate analgesia in regional anesthetic techniques by blocking voltage-gated Na+ channels and blocking transmission of neural impulses. Administration of adjuvants with local anesthetics to prolong the analgesic effects of regional anesthetic techniques has been the focus of recent studies.

Buprenorphine, as described previously, is a mu opioid receptor agonist; when used at the site of peripheral nerves, buprenorphine acts on both mu-opioid receptors in C-fiber axons and voltage-gated Na+ channels via the local anesthetic binding site [16,17]. Buprenorphine has been studied previously as a supplement to local anesthetics in both upper extremity and lower extremity regional blocks. Patil et al. studied the effects of 3 mcg/kg of buprenorphine to 0.5% bupivacaine for supraclavicular blocks for upper limb surgeries. They found that the buprenorphine group had a prolonged duration of analgesia (16.04 ± 3.19 vs. 6.20 ± 0.74 hours, p = 0.00) and sensory blockade (5.70 ± 0.94 vs. 4.94 ± 0.70 hours, p = 0.01) without any increase in side effects, including nausea, pruritis, urinary retention, or respiratory depression, when compared to bupivacaine only [18]. Similarly, Candido et al. found in a prospective randomized trial that buprenorphine with bupivacaine lowered pain scores (50% reduction in pain scores versus bupivacaine alone) and decreased opioid consumption in patients receiving infragluteal sciatic nerve blocks with bupivacaine only for foot and ankle surgery [19]. Additionally, the authors have previously demonstrated that the addition of buprenorphine to bupivacaine for ACBs significantly improved postoperative pain relief and reduced opioid consumption in TKA patients [20].

Magnesium sulfate, although it has no direct antinociceptive effects, has been shown to reduce pain and perioperative anesthetic and analgesic requirements [21]. The analgesic effect of magnesium sulfate comes from the inhibition of calcium ions from entering cells via the blocking of NMDA receptors. The specific way in which magnesium affects peripheral nerves remains unclear. Some theories suggest it may involve Na+ gating and hyperpolarization, along with antagonizing peripheral NMDA receptors [22-24]. As with buprenorphine, magnesium sulfate has been studied previously as an adjuvant to local anesthetics in both upper extremity and lower extremity blocks. Ekmekci et al. studied patients undergoing arthroscopic anterior cruciate ligament reconstruction surgery and receiving FNBs with 0.25% levobupivacaine and 150 mg magnesium sulfate in comparison to patients receiving levobupivacaine only. Pain scores were lower at four hours, six hours, 12 hours, and 24 hours in the magnesium group when compared to the control (p = 0.001). Additionally, total opioid consumption was lower in the magnesium group (391.8 ± 171.7 mg vs. 558.8 ± 357.3 mg) with no difference in side effects between the two groups [25]. These results were similar to Lee et al., who studied the addition of magnesium sulfate to bupivacaine in interscalene nerve blocks for rotator cuff repair [26]. They found that the duration of analgesia was increased in the magnesium group (553 ± 155 vs. 664 ± 188 min, p = 0.017) without any increase in side effects. In a study by Choi et al., the addition of magnesium to bupivacaine in ACBs significantly reduced opioid consumption and pain scores in TKA patients without increasing the incidence of nausea. Specifically, opioid consumption decreased significantly in the magnesium group compared to the control group over the first 24 hours (33.2 ± 3.0 vs. 21.3 ± 2.4, P = 0.003), the second 24 hours (35.4 ± 2.7 vs. 27.3 ± 2.3, P = 0.026), and the total first 48 hours after surgery (68.6 ± 5.1 vs. 48.6 ± 4.3, P = 0.004). Pain scores were also significantly reduced in the magnesium group at both 24 hours (5.1 ± 2.3 vs. 3.5 ± 2.0, P = 0.000) and 48 hours (5.1 ± 1.6 vs. 3.9 ± 1.6, P = 0.000) post surgery [27].

Meta-analyses supporting the use of both buprenorphine and magnesium as adjuvants in peripheral nerve blocks have also been recently completed. Schnabel and colleagues studied buprenorphine and included 12 randomized controlled trials [16]. They found that patients receiving buprenorphine with local anesthetics reported a longer duration of analgesia when compared with patients receiving local anesthetic alone (mean difference (MD): 8.64 hours, 95% CI: 6.44-10.85); p < 0.01), but they noted that the group receiving buprenorphine had a higher relative risk (RR) for developing postoperative nausea and vomiting (RR: 5.0, 95% CI: 1.12-22.27; p = 0.03). In 2021, Zeng et al. examined the use of magnesium sulfate in peripheral nerve blocks. They analyzed 21 randomized controlled trials [22]. The authors found that magnesium sulfate increased the total duration of sensory blockade (MD: 114.59 min, 95% CI: 89.31-139.88, p < 0.00001), reduced VAS scores at six hours (MD: 1.36 points, 95% CI: -2.09 to -0.63; p = 0.0003) and 12 hours (1.54 points, 95% CI: -2.56 to -0.53; p = 0.003), and reduced postoperative analgesic use at 24 hours post surgery (MD: -2.06, 95% CI: -2.76 to -1.35; p < 0.00001). The authors added that the incidence of nausea and vomiting was also reduced by 44% in patients receiving adjuvant magnesium sulfate (95% CI: 0.35-0.92; p = 0.02).

There has been considerable research involving both of our studied adjuvants for regional anesthesia. However, the majority of studies compare the addition of various adjuvants to local anesthetics alone, generally demonstrating improvements in pain management with adjuvants as previously discussed. There are few studies comparing individual adjuvants plus local anesthetics to each other. To date, no studies have compared magnesium and buprenorphine in regional blocks, particularly in the context of SDD TKA.

Our study is the first randomized controlled trial investigating the effects of magnesium versus buprenorphine in ACB for SDD TKA. We found that both adjuvants were comparable in their effect on patient pain scores and opioid consumption at both 24 and 48 hours post surgery. Both groups showed high levels of satisfaction, which was likely influenced by a multitude of factors such as age, sex, patient personality, expectations, physical and psychological comorbidities, severity of arthropathy, anesthesia, postoperative pain management, surgical technique, implant type, and postoperative rehabilitation. Notably, the occurrences of nausea and vomiting were similar in both groups, as were the levels of overall satisfaction.

Despite these promising results, our study has several limitations. First, the lack of a placebo group leaves ambiguity about the potential risks of both drugs. Second, while we enrolled a comparable number of patients to other similar studies, a larger sample size might have revealed additional subtle differences that our current study did not capture. For instance, we observed a reduction in average opioid consumption among women on postoperative day two in the Mag group in comparison to the Bup group; however, this reduction was not statistically significant and might have reached significance with a larger sample size. Additionally, we only asked for VAS scores at specific times to simplify reporting, but this single report per day may not fully reflect pain experienced throughout the day. Finally, we did not measure serum levels of our adjuvant medications, which could have helped to isolate the peripheral versus central effects of each drug.

## Conclusions

In conclusion, magnesium and buprenorphine were comparable in their effects on postoperative pain and opioid consumption at 24 and 48 hours following surgery, with similar incidences of nausea and vomiting. Both agents, due to their favorable benefits and low side effect profiles, can serve as practical and effective adjuncts for regional anesthesia in TKA. Given their similar efficacy, magnesium sulfate may represent a more cost-effective option, as its unit cost was approximately one-third that of buprenorphine ($4.16 vs. $12.11) at our institution. Further research is warranted to clarify their peripheral versus central mechanisms of action and to evaluate their influence on long-term outcomes.

## Figures and Tables

**Figure 8:**
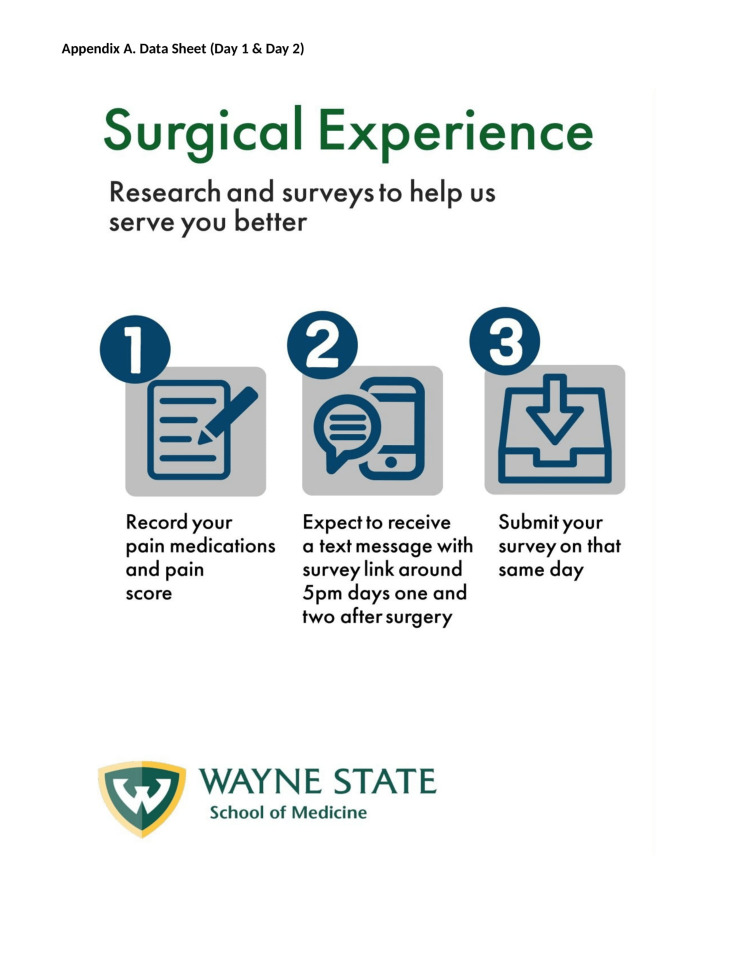
Surgical experience - page 1.

**Figure 9:**
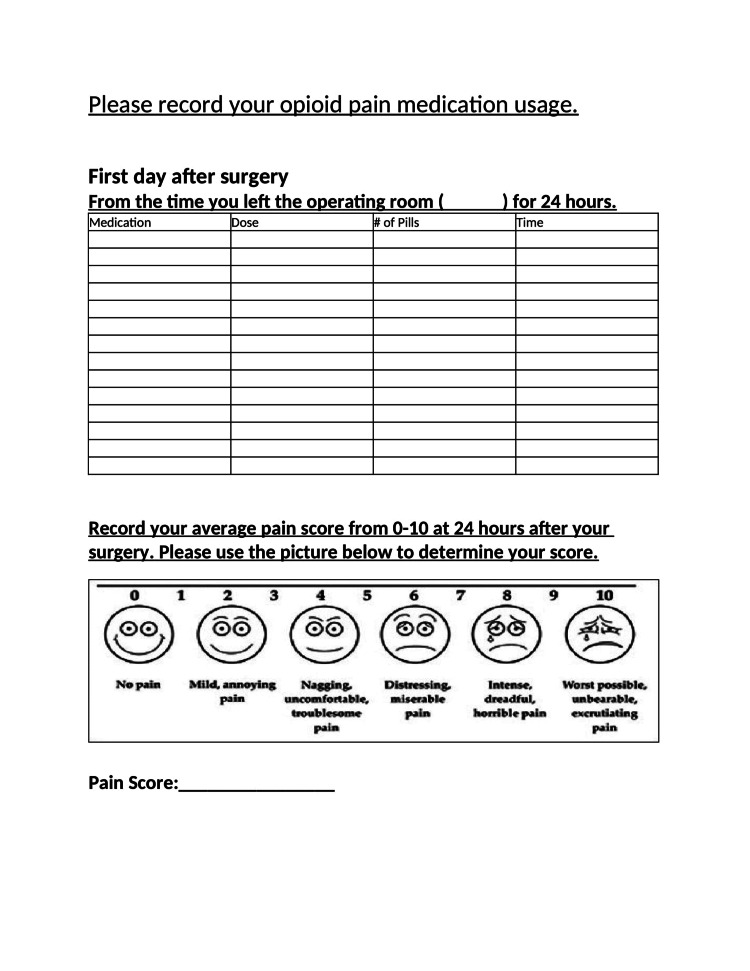
Surgical experience - page 2.

**Figure 10:**
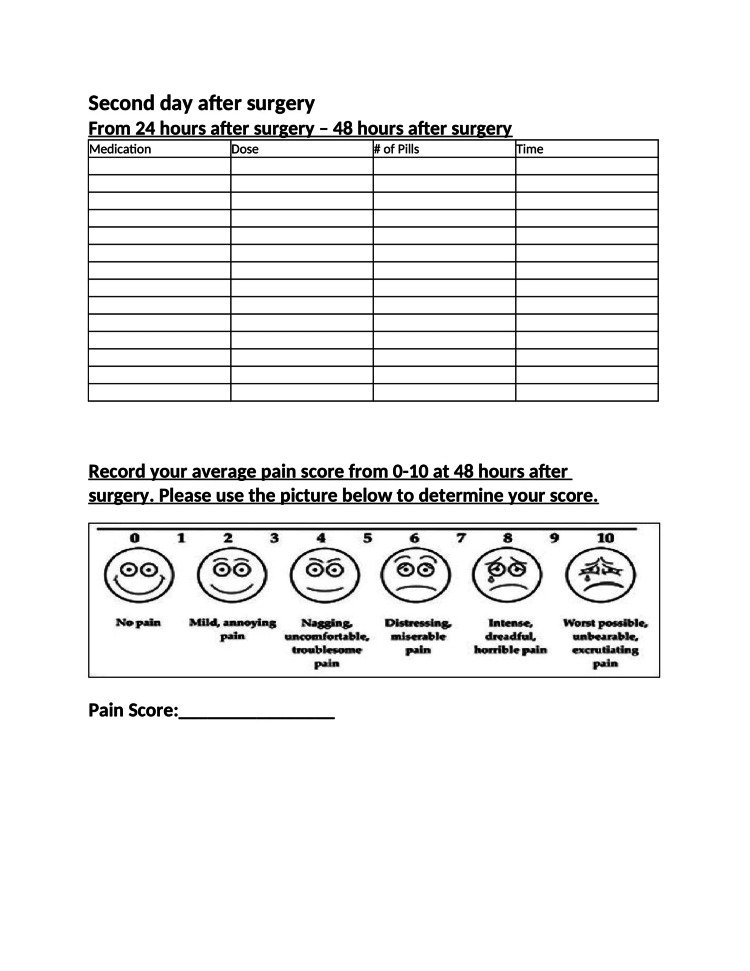
Surgical experience - page 3.

**Figure 11:**
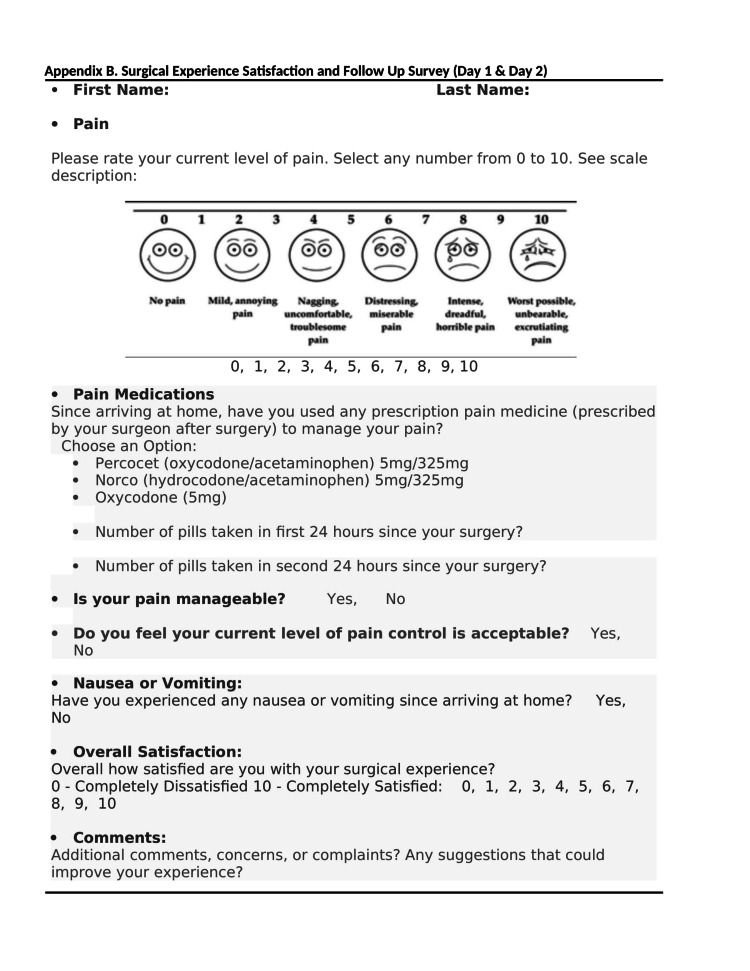
Survey.

## Appendices

Appendix A

Appendix B
